# Endocannabinoid System and Cannabinoid 1 Receptors in Patients With Pharmacoresistant Temporal Lobe Epilepsy and Comorbid Mood Disorders

**DOI:** 10.3389/fnbeh.2020.00052

**Published:** 2020-05-06

**Authors:** Luisa Rocha, Resat Cinar, Rosalinda Guevara-Guzmán, Mario Alonso-Vanegas, Daniel San-Juan, Iris Martínez-Juárez, José Luis Castañeda-Cabral, Francia Carmona-Cruz

**Affiliations:** ^1^Department of Pharmacobiology, Center of Research and Advanced Studies, Mexico City, Mexico; ^2^Laboratory of Physiologic Studies, National Institute on Alcohol Abuse and Alcoholism, National Institutes of Health, Rockville, MD, United States; ^3^Department of Physiology, School of Medicine, National Autonomous University of Mexico, Mexico; ^4^National Institute of Neurology and Neurosurgery “Manuel Velasco Suarez”, Mexico City, Mexico

**Keywords:** mesial temporal lobe epilepsy, anandamide, oleoylethanolamine, 2-arachidonoylglycerol, cannabinoid 1 receptor, anxiety, depression

## Abstract

Experimental evidence points out that the activation of the endocannabinoid system induces neuroprotective effects and reduces mood disorders. In the hippocampus of patients with mesial temporal lobe epilepsy (MTLE), studies indicated augmented cannabinoid 1 receptor (CB_1_R) binding, in spite of its low mRNA and protein expressions. Although this situation suggests an enhanced CB_1_R-induced neurotransmission in patients with MTLE, especially those with pharmacoresistant seizures, which present important neuronal damage and high comorbid mood disorders. The present study focused to investigate the status of CB_1_R and the endocannabinoid system by obtaining CB_1_R-induced G-protein signaling efficacy and measuring the tissue levels of endocannabinoids in the hippocampus and the temporal neocortex of patients with pharmacoresistant MTLE. Furthermore, the obtained results were correlated with comorbid anxiety and depression. The experiments revealed that patients with MTLE present increased CB_1_R-induced G-protein signaling efficacy (Emax) as well as an augmented tissue content of anandamide and oleoylethanolamine and low 2-arachidonoylglycerol. Some of these changes were more evident in patients with MTLE without mood disorders. The current findings indicate that pharmacoresistant MTLE is associated with increased CB_1_R-induced transductional mechanisms as well as augmented tissue content of specific endocannabinoids in the hippocampus and the temporal neocortex. The enhanced endocannabinoid neurotransmission may be involved in the absence of comorbid mood disorders in some patients with MTLE.

## Introduction

Endocannabinoids are synthesized “on demand” as a consequence of enhanced neuronal depolarization and elevated intracellular calcium levels (Stella et al., 1997). According to this notion, it is expected that the augmented neuronal depolarization produced during a seizure activity may result in the activation of the endocannabinoid system. This notion is supported by experimental evidence indicating that the tissue levels of endocannabinoids 2-arachidonoylglycerol (2-AG) and anandamide (AEA) were augmented subsequent to the induction of acute seizures and induce neuroprotective effects through the activation of cannabinoid 1 receptors (CB_1_Rs) (Marsicano et al., 2003; Wallace et al., 2003). However, drug-naive patients with temporal lobe epilepsy (TLE), a disorder associated with enhanced glutamatergic neurotransmission during ictal and interictal periods (During and Spencer, 1993), present reduced AEA levels in CSF, whereas the 2-AG levels are not modified (Romigi et al., 2010).

Concerning endocannabinoid receptors, studies revealed reduced mRNA and protein expression of CB_1_Rs in the hippocampus of patients with pharmacoresistant TLE, especially in glutamatergic axon terminals (Ludányi et al., 2008). In contrast, positron emission tomography (PET) imaging experiments using [^18^F]MK-9470 indicate that patients with mesial TLE (MTLE) present augmented CB_1_R availability in the temporal lobe ipsilateral to the epileptic focus (Goffin et al., 2011). These studies indicate that, in spite of their low mRNA and protein expression, CB_1_R binding is enhanced in the brain of patients with MTLE. This situation leads to propose an enhanced CB_1_R-induced neurotransmission with subsequent inhibitory and neuroprotective effects in brains with epilepsy. However, this notion is not supported by the progressive and severe hippocampal damage found in patients with pharmacoresistant MTLE that suggests a hypoactive endocannabinoid neurotransmission (Nearing et al., 2007).

A deficient endocannabinoid neurotransmission is also associated with anxiety and depression (Boorman et al., 2016; Korem et al., 2016; Kranaster et al., 2017), whereas the augmentation of endocannabinoids is related with antidepressant effects (Bortolato et al., 2007). Considering that pharmacoresistant MTLE frequently coexists with anxiety and depression (Nogueira et al., 2017), it is possible to suggest that the hypoactivity of the endocannabinoid system may play a significant role in their comorbidity. However, no evidence exists to support this notion.

The present study focused to establish that MTLE is associated with alterations in the endocannabinoid system that facilitate the seizure activity and the comorbid anxiety and depression. Experiments were designed to evaluate the tissue content of endocannabinoids and the transductional mechanisms subsequent to the activation of CB_1_Rs in the hippocampus and the temporal neocortex of patients with pharmacoresistant MTLE, with and without anxiety and depression (A/D).

## Materials and Methods

### Patients and Tissue Collection

Hippocampus and temporal neocortex tissues were obtained from 49 patients (29 females and 20 males) with pharmacoresistant MTLE. Every patient underwent an extensive presurgical evaluation, including video electroencephalogram (EEG), magnetic resonance imaging (MRI), and single photon emission computed tomography (SPECT) within the Epilepsy Surgery Program of the National Institute of Neurology and Neurosurgery (Instituto Nacional de Neurologia y Neurocirugia “Manuel Velasco Suarez,” Mexico). Scalp EEG played an important role in lateralizing and focalizing interictal epileptiform activity. Video-EEG was performed, and at least two complex partial seizures were recorded in each patient. Since we could not perform ictal SPECT in each patient, interictal SPECT offered valuable information regarding the hypoperfusion area (Tae et al., 2005; Huberfeld et al., 2006). MRI performed with either a 1.5- or 3-T machine demonstrated mesial sclerosis and the reduced volume of the temporal pole area of epileptic patients with MTLE, but no significant changes in T2–T3 gyri from all epileptic patients. In addition, MRI findings showed a clear matching with the EEG recordings. Patients with focal cortical dysplasia or neocortical temporal lobe epilepsy were specifically excluded from the study.

During the neurological evaluation, the prevalence of depression and anxiety disorders was established using the Structured Clinical Interview for DSM-IV Axis I (First et al., 1999). A Spanish version of the Hospital Anxiety and Depression Scale (HADS), previously validated in a Spanish population (Herrero et al., 2003; Gómez-Arias et al., 2012), was applied to all patients to identify symptoms of anxiety and/or depression. The HADS scale considers symptoms over the previous week and is not affected by coexisting general medical conditions. Patients with other psychiatric or somatic disturbances interfering with mood disorders, such as addiction, were excluded from the present study. This study was approved by the scientific committees of the institutions involved in the present research, and informed consent was obtained from every patient.

The patients had “en block” anterior lobectomy, ipsilateral to the epileptic focus, at least 48 h after the last seizure. Intraoperative electrocorticography was performed with grids of 4 × 8 electrodes (Ad-Tech, Racine, WI, United States) in order to identify spiking neocortex. Samples from both the epileptic hippocampus and the spiking T2–T3 gyri (from 2.5 to 5 cm posterior to the temporal pole) were obtained in every patient. Tissue was collected immediately upon resection, quickly frozen in pulverized dry ice, and stored at −70°C.

It is known that endocannabinoid AEA and another *N*-acyl ethanolamine oleoylethanolamide (OEA) present a progressive accumulation after death, a condition that correlates with the postmortem interval (Patel et al., 2005; Schmid et al., 2005). Then, biopsies from the hippocampus and the temporal neocortex obtained from seven patients (three males and four females) who had a cerebral lesion without epilepsy (four with tumor and three with vascular malformation) were used as control condition for endocannabinoid tissue content. These patients had to have a surgical resection of a portion of these brain areas. As control condition for the binding experiments, we used autopsy samples obtained from 11 subjects (seven males and four females) who died by vehicular accident (*n* = 6), cardiac arrest (*n* = 4), pneumonia (*n* = 1), and without history of neurological disease. These autopsy samples were obtained with a postmortem interval of 14.8 ± 0.9 h and immediately stored at −70°C. The fragments from the neocortex included gray matter only.

### Evaluation of Endocannabinoid Tissue Content

Endocannabinoids AEA, 2-AG, and OEA were quantified in the brain by liquid chromatography/tandem mass spectrometry as previously described (Cinar et al., 2014). Briefly, brain tissue weighing 100–150 mg was homogenized in 0.5 ml of ice-cold methanol/Tris buffer (50 mM, pH 8.0), 1:1, containing 11.2 ng [^2^H_4_]AEA as internal standard. The homogenates were extracted three times with CHCl_3_: MeOH (2:1, vol/vol), dried under nitrogen flow, and reconstituted with MeOH after precipitating proteins with ice-cold acetone. The mass spectrometer was set for electrospray ionization operated in positive ion mode. The levels of each compound were analyzed by multiple reactions monitoring. The molecular ion and fragment for each compound were measured as follows: m/z 348.3→62.1 for AEA, m/z 352.3→66.1 for [^2^H_4_]AEA, m/z 326.3→62.1 for OEA, and m/z 379.3→91.1 for 2−AG. The analytes were quantified using MassHunter Workstation LC/QQQ Acquisition and MassHunter Workstation Quantitative Analysis software (Agilent Technologies). The amount of AEA, 2-AG, and OEA in the samples was determined against standard curves. Values are expressed as fmol/mg (AEA and OEA) or pmol/mg (2-AG), respectively.

### Analysis of Gi/o Protein Activation by CB1Rs

#### Membrane Preparations

Crude membrane fraction from human temporal neocortex and hippocampus was prepared according to the method previously described (Benyhe et al., 1997). Briefly, samples (50–100 mg) were homogenized on ice in centrifugation buffer (50 mM Tris HCl, 1 mM EGTA, and 3 mM MgCl_2_; pH 7.4) using a Teflon glass homogenizer. The homogenate was centrifuged at 20,000 × *g* for 45 min at 4°C, and the resulting pellet was resuspended in assay buffer (50 mM Tris HCl, 9 mM MgCl_2_, 0.2 mM EGTA, and 150 mM NaCl; pH 7.4). The centrifugation step was repeated. The final pellet was resuspended in assay buffer and homogenized. Protein levels were determined by the method of Lowry (Lowry et al., 1951). The sample was diluted to a concentration of 2 μg/ml with assay buffer and stored at −70°C until use in the binding assays.

#### [^35^S]GTPγS Binding Assay

Receptor-mediated Gi/o protein activation was measured as described previously (Cinar et al., 2008) with slight modifications. Cell membrane fractions (≈10 μg of protein/sample) were incubated at 30°C for 60 min in assay buffer containing 0.1% fatty acid-free bovine serum albumin with GDP (100 μM), [^35^S]GTPγS (0.05 nM), and increasing concentrations (10^–9^ to 10^–5^ M) of WIN 55212-2 in assay tubes with a final volume of 1 ml. Total binding was measured in the absence of the tested compound. Non-specific binding was determined in the presence of 100 μM unlabeled GTPγS and subtracted from the total binding to calculate the specific binding. The reaction was initiated with incubation at 30°C for 60 min and terminated by the addition of ice-cold wash buffer (50 mM Tris HCl and 5 mM MgCl_2_; pH 7.4), followed by rapid filtration under vacuum through Whatman GF/B glass-fiber filters. The filters were washed three times with ice-cold wash buffer using Brandel M48 Cell Harvester and then dried, and bound radioactivity was detected in Sigma-Fluor^TM^ Scintillation Cocktail (Sigma) with Beckman LS6000-SC liquid scintillation counter. Stimulation was established as percent of the specific [^35^S]GTPγS binding observed in the absence of receptor ligands (basal activity). [^35^S]GTPγS binding experiments were performed in triplicates and repeated at least three times. Data were subjected to non-linear regression analysis of concentration effect curves performed by Prism (GraphPad Software, Inc.) to determine the potency (EC_50_) and the maximal stimulation (Emax) values.

### Data Analysis

The results were examined statistically by one-way ANOVA and a *post hoc* Tukey test to determine significant differences. Pearson correlation calculations were carried out to identify the influence of clinical factors (age of patients, age at seizure onset, epilepsy duration, and seizure frequency) on the results obtained. Data were expressed as mean ± SME.

## Results

### Clinical Data

Patients with MTLE without A/D (*n* = 25) had the following clinical data (mean ± SE): age of subjects, 30.7 ± 1.8 years; age at seizure onset, 10.5 ± 2 years; epilepsy duration, 19.2 ± 2.2 years; and frequency of seizures, 8.5 ± 1.5 per month. Patients with MTLE and comorbid A/D (*n* = 24) presented similar clinical data (age of subjects, 37.9 ± 1.7 years; age at seizure onset, 10.9 ± 1.4 years; and frequency of seizures, 13.8 ± 4.2 per month) when compared with patients without A/D, except that they presented a longer epilepsy duration (27.1 ± 2.1 years, *p* < 0.02). The age of the patients with cerebral lesion without epilepsy and the autopsy subjects was not significantly different from that of the patients with MTLE (35.2 ± 7.7 and 42.9 ± 6.1 years, respectively; *p* > 0.05).

### Endocannabinoids in the Hippocampus and the Temporal Neocortex of Patients With Pharmacoresistant Mesial Temporal Lobe Epilepsy

The tissue levels of endocannabinoids and OEA in the hippocampus and the temporal neocortex of control subjects showed the following values: AEA, 26.8 ± 2.1 and 17.8 ± 1.8 fmol/mg, respectively; 2-AG, 4378 ± 941 and 2620 ± 423 pmol/mg, respectively; and OEA, 208.9 ± 29 and 127 ± 18 fmol/mg, respectively.

**FIGURE 1:**
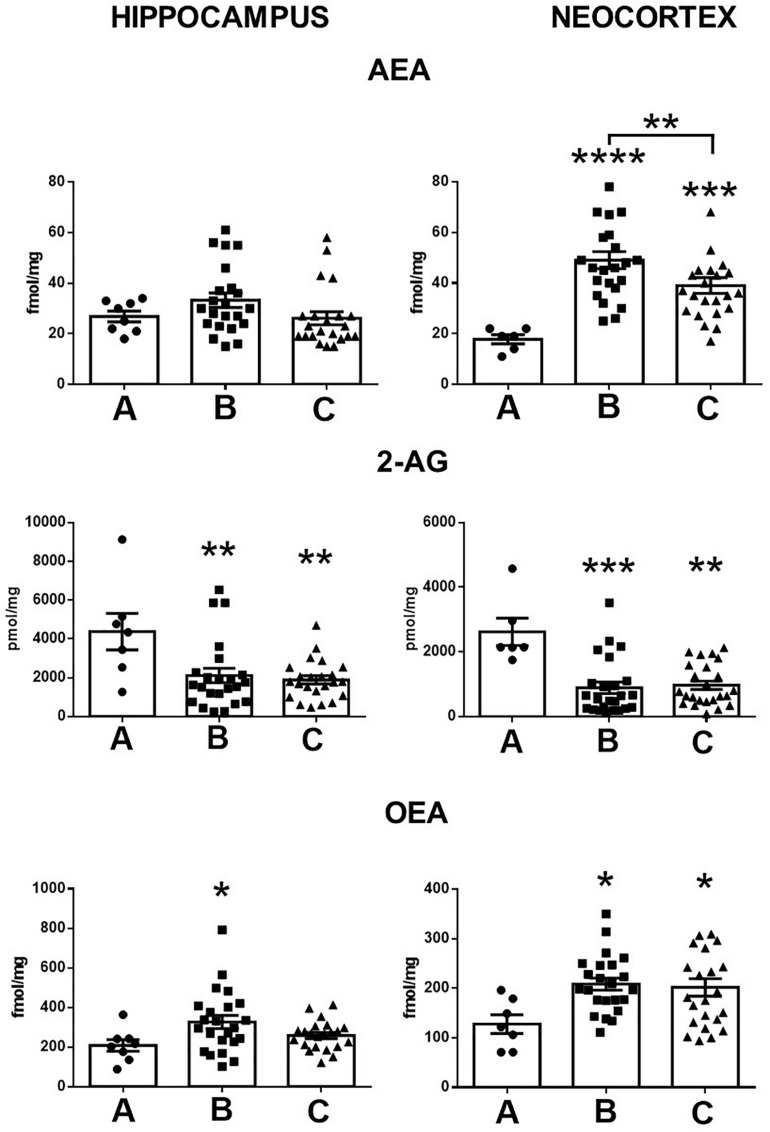
Tissue content of anandamide (AEA), 2-arachidonoylglycerol (2-AG), and oleoylethanolamine (OEA) in the hippocampus and the temporal neocortex of autopsies **(A)** and patients with mesial temporal lobe epilepsy without **(B)** and with comorbid anxiety and depression **(C)**. Values are expressed as mean ± SE. *p < 0.05; **p < 0.01; ***p < 0.001; ****p < 0.0001.

When compared with the control conditions, the hippocampus of patients with MTLE without mood disorders showed a high tissue content of OEA (57%, *p* < 0.04) and low 2-AG tissue levels (51%, *p* < 0.005) (Figure 1). The temporal neocortex of these patients presented a high tissue content of AEA and OEA (175%, *p* < 0.001 and 63%, *p* < 0.02, respectively) and a low tissue content of 2-AG (65%, *p* < 0.0001).

In contrast with the control condition, the hippocampus of patients with MTLE plus A/D presented low tissue levels of 2-AG (53%, *p* < 0.002). In the temporal neocortex, experiments revealed a high tissue content of AEA and of OEA (118%, *p* < 0.008 and 58%, *p* < 0.04, respectively) and a low tissue content of 2-AG (62%, *p* < 0.0001).

In contrast to patients with A/D, the tissue levels of AEA in the temporal neocortex of patients with MTLE without comorbid alterations were significantly higher (25%, *p* < 0.02). No further significant differences were found between both groups of patients. In addition, statistical analysis did not reveal significant correlations between the tissue content of endocannabinoids and the clinical factors (Table 1).

**TABLE 1:** Correlations between clinical data and parameters evaluated in hippocampus and temporal neocortex of patients with mesial temporal lobe epilepsy with and without mood disorders.

Parameters	Patients	Brain area	Age of patient	Seizure onset age	Duration of epilepsy	Frequency of seizures
**AEA**	**A/D**	**Hipp**	–0.1500	0.1356	–0.2140	–0.0404
		**Cx**	–0.1104	0.1527	–0.1988	–0.0009
	**No A/D**	**Hipp**	–0.0375	0.1084	–0.2451	–0.1648
		**Cx**	–0.0560	0.1175	–0.2515	–0.1510
**2-AG**	**A/D**	**Hipp**	0.3291	–0.1964	0.4300	–0.0041
		**Cx**	0.3405	–0.0656	0.3212	–0.2224
	**No A/D**	**Hipp**	–0.0819	0.0909	–0.1177	–0.1361
		**Cx**	0.2408	–0.1382	0.3074	–0.0203
**OEA**	**A/D**	**Hipp**	–0.1032	0.0891	–0.1426	–0.0823
		**Cx**	–0.0595	0.0931	–0.1181	0.0135
	**No A/D**	**Hipp**	–0.0661	–0.0222	–0.0961	–0.0664
		**Cx**	–0.0544	0.0300	–0.1926	–0.0635
**Emax CB_1_Rs**	**A/D**	**Hipp**	0.3659	0.0233	0.2367	0.0191
		**Cx**	0.0305	0.5300	–0.4265	0.0474
	**No A/D**	**Hipp**	–0.4980	–0.3488	–0.0406	–0.0726
		**Cx**	–0.0362	0.4122	–0.5234	–0.2319

*Values represent the Pearson Correlation Coefficients. A/D, patients with mesial temporal lobe epilepsy with anxiety and/or depression; AEA, anandamide; CB_1_Rs, cannabinoid 1 receptor; Cx, neocortex; Emax, G-protein signaling efficacy; Hipp, hippocampus; No A/D, patients with mesial temporal lobe epilepsy without anxiety and/or depression; OEA, oleoylethanolamine; 2-AG, 2-arachidonoylglycerol.*

### Gi/o Protein Activation by CB_1_Rs in Patients With Mesial Temporal Lobe Epilepsy

**FIGURE 2:**
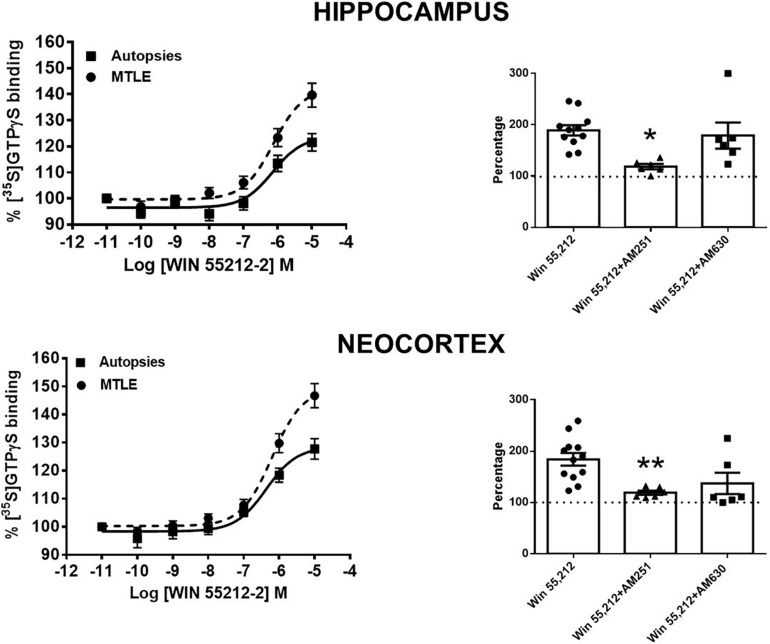
Left side: specific [^35^S]GTPγS binding to cell membranes obtained from the hippocampus and the temporal neocortex of autopsies and patients with mesial temporal lobe epilepsy (MTLE) as a function of increasing concentrations of WIN 55212-2. Each point represents the mean ± SME of the individual percentage stimulation over the basal values. The absolute basal values from patients with MTLE were similar to those from the autopsy samples. Notice that in patients with MTLE, the [^35^S]GTPγS binding stimulation by WIN 55212-2 was higher with respect to autopsies. Right side: representation of the maximal stimulation (Emax) values induced by WIN 55212-2 alone and in the presence of an antagonist of CB1Rs (AM251) or CB2Rs (AM630) in the hippocampus and the temporal neocortex of patients with MTLE. Notice that AM251 avoided the augmentation of Emax in both brain areas. Values are expressed as mean ± SE of the individual percentage stimulation over basal values (dotted lines). *p < 0.05; **p < 0.01.

In autopsy samples, the binding assay in the presence of WIN 55212-2 revealed a maximal incorporation of [^35^S]GTPγS (Emax) of 25.5 and 29.6% (hippocampus and neocortex, respectively), with EC_50_ values of 704 ± 127 and 378 ± 64 nM (hippocampus and temporal neocortex, respectively). When compared with the autopsy samples, the tissue from patients with MTLE showed higher Emax values in both the hippocampus (14%, *p* < 0.01) and the temporal neocortex (17%, *p* < 0.01), whereas the EC_50_ values were similar (511 ± 110 and 637 ± 80 nM, respectively) (Figure 2).

**FIGURE 3:**
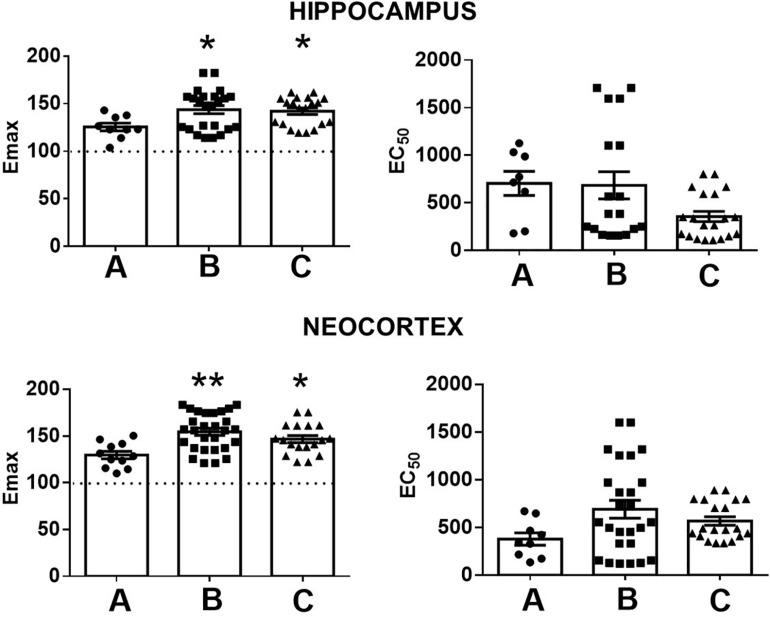
Representation of the maximal stimulation (Emax) and potency (EC_50_) values induced by WIN 55212-2 in the hippocampus and the temporal neocortex of autopsies **(A)** and patients with mesial temporal lobe epilepsy without **(B)** and with comorbid anxiety and depression **(C)**. Values are expressed as mean ± SE. *p < 0.05; **p < 0.01.

A comparison of Emax and EC_50_ values according to the presence or the absence of comorbid A/D maintained these significant differences with the autopsies. However, this analysis revealed a higher Emax value in the temporal neocortex of patients with MTLE without A/D. No significant differences were detected between both groups of patients with MTLE (Figure 3). In addition, no significant correlations were detected between the Emax values and the clinical factors (Table 1).

These results indicate that WIN 55212-2, an agonist for CB_1_R and CB_2_R (Eissenstat et al., 1995), induces a higher Gi/o protein activation in the tissue obtained from patients with MTLE. In order to identify the contribution of CB_1_R and/or CB_2_R in this effect, Emax values were obtained in the presence of AM251 and AM630 (antagonists of CB_1_R and CB_2_R, respectively) at 100 μM. The results obtained revealed that AM251 attenuated the WIN 55212-2-induced efficacy (Emax) in both the hippocampus and the temporal neocortex of patients with MTLE. This effect was not evident with AM630 (Figure 2). These findings support that the higher Gi/o protein activation induced by WIN 55212-2 in the tissue of patients with MTLE was mediated by CB_1_Rs.

## Discussion

The present study revealed a higher CB_1_R-induced Gi/o protein activation and significant changes in the tissue content of AEA, OEA, and 2-AG in the epileptic hippocampus and the temporal neocortex of patients with pharmacoresistant MTLE. Some of these changes were more evident in patients without comorbid A/D.

CB_1_Rs are involved in the modulation of glutamatergic and GABAergic transmission in the hippocampus and the neocortex (Hoffman et al., 2003; Domenici et al., 2006; Kawamura et al., 2006; Hill et al., 2007). The effects of CB_1_Rs depend on their location, i.e., increased CB_1_R signaling on glutamatergic terminals induces inhibition and neuroprotective effects, while those located on GABAergic terminals induce excitatory effects (Chiarlone et al., 2014; Guggenhuber et al., 2015). Studies support that the endocannabinoid system induces protective effects in several neurological disorders (Kaur et al., 2016). In neuropathic pain, CB_1_Rs along with endocannabinoids are augmented, a situation explained as a compensatory condition (Mitrirattanakul et al., 2006).

In the present study, we found that WIN 55,212-2, a potent agonist for both CB_1_R and CB_2_R (Eissenstat et al., 1995), induced an overactivation of Gi/o proteins in both the hippocampus and the temporal neocortex of patients with pharmacoresistant MTLE. According to the obtained results, it is possible that the WIN 55,212-2-induced overactivation of Gi/o protein was mediated by CB_1_R activation. The increased CB_1_R-induced Gi/o protein activation found in the present study correlates with the high CB_1_R binding detected by PET in the temporal lobe of patients with MTLE (Goffin et al., 2011). This suggests an increase of neurotransmission mediated by CB_1_Rs in these brain areas, in spite of the lower mRNA and protein expressions of CB_1_R (Ludányi et al., 2008).

The functional consequence of the increased CB_1_Rs-induced Gi/o protein activation in the present study is that endocannabinoid neurotransmission is augmented at brain areas involved in MTLE. This notion was supported by previous studies in which cannabinoid agonists were more effective in suppressing recurrent excitation in the dentate gyrus of animals with augmented expression of CB_1_Rs subsequent to epileptic activity rather than in the controls (Bhaskaran and Smith, 2010).

Epilepsy induces a significant reorganization of the CB_1_Rs (Falenski et al., 2007). In the hippocampus of patients with pharmacoresistant MTLE, there is a reduction in the number of excitatory synapses, an effect associated with a low expression of CB_1_Rs (Ludányi et al., 2008), whereas the inhibitory presynaptic terminals present a high expression of CB_1_Rs (Maglóczky et al., 2010). In addition, the hippocampus of patients with MTLE presents reactive astrogliosis, a condition in which endocannabinoid neurotransmission can augment the glutamate release and then promote the seizure activity (Navarrete and Araque, 2010; Coiret et al., 2012). In the neocortex of patients with pharmacoresistant epilepsy, the activation of CB_1_Rs inhibits GABA_A_ receptor-mediated synaptic transmission (Kovacs et al., 2012). Then, it is possible to suggest that the increased CB_1_R-induced Gi/o protein activation found in the hippocampus and the temporal neocortex of patients with MTLE reduces the seizure threshold and induces proconvulsant effects.

The blockage or genetic disruption of CB_1_Rs induces depression and anxiogenic effects (Navarro et al., 1997; Haller et al., 2004; Christensen et al., 2007; Mikics et al., 2009). An important finding from the present study was that the enhanced CB_1_R-induced Gi/o protein activation was similar in patients with MTLE with and without comorbid A/D. These results support that the augmentation in CB_1_R-induced transductional mechanisms in the hippocampus and the temporal neocortex of patients with MTLE is not involved in comorbid A/D. However, additional studies are necessary to support this hypothesis.

Concerning endocannabinoid tissue levels, the results of the present study revealed that patients with MTLE present opposite changes, i.e., high levels of AEA and OEA and decreased levels of 2-AG. This condition was more evident in the temporal neocortex. Changes in the opposite direction have been found in the brain areas of patients with schizophrenia (Muguruza et al., 2013). They can be explained as a consequence of the distinct metabolism and the catabolism of each endocannabinoid (Di Marzo and Maccarrone, 2008). In addition, studies support that the augmentation of AEA reduces the levels, metabolism, and effects of 2-AG, an effect mediated by TRPV1 channels (Maccarrone et al., 2008).

AEA and 2-AG are endogenous ligands for CB_1_Rs (Hillard, 2000) with anticonvulsant and neuroprotective effects (Wallace et al., 2002; Marsicano et al., 2003; Vilela et al., 2013; Mounsey et al., 2015; Sugaya et al., 2016). The low tissue levels of 2-AG detected in the hippocampus and the temporal neocortex of patients with MTLE can be the consequence of the low expression of the enzyme responsible for its synthesis (diacylglycerol lipase) as found in these subjects (Ludányi et al., 2008). Regarding the AEA, a previous study indicated that the hippocampus of patients with MTLE do not present alterations in the tissue content of this endocannabinoid, a condition associated with the absence of changes in its synthesis and metabolism (Ludányi et al., 2008). The present study supports the absence of alterations in the tissue levels of AEA in the epileptic hippocampus. In contrast, our results revealed an increase of AEA tissue content in the temporal neocortex, a condition that can be associated to the excessive glutamatergic neurotransmission related with epileptic activity (During and Spencer, 1993; Hansen et al., 2001).

It is important to notice that the changes in OEA tissue levels found in the present study were similar to those detected for AEA. This finding can be explained because OEA is a structural analog of AEA. Their synthesis and degradation are controlled by the same enzymes such as *N*-acyl phosphatidylethanoplamine specific phospholipase D and fatty acid amide hydrolase, respectively. It is known that OEA induces anti-inflammatory, neuroprotective, and anti-depressant effects (Antón et al., 2017). Then, the increased tissue content of OEA in both the hippocampus and the temporal neocortex of patients with pharmacoresistant MTLE may represent a mechanism to reduce cell damage.

Experimental evidence indicates that enhanced levels of endocannabinoids induce antidepressive and anxiolytic effects (Kathuria et al., 2003; Hill and Gorzalka, 2005; Yu et al., 2015; Brellenthin et al., 2017; Kranaster et al., 2017; Meyer et al., 2019). Subjects with major depression show low serum levels of endocannabinoids (Hill et al., 2008, 2009), whereas impaired 2-AG signaling in the hippocampus facilitates an anxiety-like behavior (Guggenhuber et al., 2015). In contrast, other studies suggest that the increased endocannabinoid neurotransmission is associated with mood disorders (Vinod et al., 2005). Our results revealed that patients with MTLE without A/D present higher tissue content of OEA in the hippocampus, whereas AEA is augmented in the temporal neocortex, when compared with patients with MTLE and comorbid A/D. The high tissue content of OEA in the hippocampus is in agreement with a previous study indicating that its oral administration induces antidepressant effects associated with the regulation of brain-derived neurotrophic factor levels in the hippocampus (Jin et al., 2015). On the other hand, decreased levels of AEA are associated with depression (Vinod et al., 2012) and anxiogenic effects (Rubino et al., 2008), whereas its augmentation reverses depressive-like responses through the activation of CB_1_Rs (de Morais et al., 2016). It is possible that the higher levels of AEA in the temporal neocortex and OEA in the hippocampus of patients with MTLE avoid the comorbid A/D. Additional studies are essential to support this notion.

An important limitation of the present study is the lack of correlation between the results obtained and the conditions that may modify the endocannabinoid system in both autopsies and patients with MTLE. Concerning this issue, it is known that the endocannabinoid system is modified by antiseizure drugs, diets rich in fats and sugars, weight changes, herbal remedies, chronic stress, exercise, and cannabis consumption, among other conditions (McPartland et al., 2014). Unfortunately, these conditions are not considered as criteria for diagnosing and they are not rigorously investigated in autopsies and patients with epilepsy. Further clinical trials are essential to determine if the changes found in the present study are mediated by conditions different from epileptic activity.

## Data Availability Statement

The datasets generated for this study are available on request to the corresponding author.

## Ethics Statement

The studies involving human participants were reviewed and approved by Ethics Committee of the National Institute of Neurology and Neurosurgery. The patients/participants provided their written informed consent to participate in this study.

## Author Contributions

LR designed the study and organized the manuscript. RC carried out the experiments to determine the tissue levels of endocannabinoids. RG-G obtained and evaluated the autopsy samples. MA-V did the neurosurgery of patients. DS-J and IM-J identified and evaluated the patients with epilepsy. JC-C participated in the analysis of the results and organization of the manuscript. FC-C carried out the binding experiments.

## Conflict of Interest

The authors declare that the research was conducted in the absence of any commercial or financial relationships that could be construed as a potential conflict of interest.
